# Supplementary material for: Ocean iron cycle feedbacks decouple atmospheric CO2 from meridional overturning circulation changes
Source: Nat Commun. 2024 Jul 8;15:5532. doi: 10.1038/s41467-024-49274-1 (PMC11231327; doi:10.1038/s41467-024-49274-1)
Supplement: Supplementary file 1 — Supplementary Information [file 41467_2024_49274_MOESM1_ESM.pdf]

Ocean iron cycle feedbacks decouple atmospheric CO_2 from meridional overturning circulation changes

Jonathan Maitland Lauderdale[1*]

[1*]Department of Earth, Atmospheric and Planetary Sciences, Massachusetts Institute of Technology, 77 Massachusetts Avenue, Cambridge, 02139, MA, USA..

Corresponding author(s). E-mail(s): jml1@mit.edu;

Supplementary Information

Several sensitivity experiments were run to examine the robustness of the MOC-atmospheric pCO_2 relationship with fixed and variable ligand parameterizations.

Southern Ocean surface iron input

Changes in external aeolian dust deposition to the Southern Ocean are hypothesized to significantly increase iron supply during glacial periods, stimulating iron-limited biological production in the Southern Ocean to consume unused nutrients and trap CO_2 in the ocean more efficiently [1]. Conversely, more humid conditions possibly projected for the future climate reduce the aeolian iron flux [2] and could lead to enhanced ocean CO_2 outgassing [3]. To examine the effect of variations in external Southern Ocean iron input on the relationship between changes in MOC and atmospheric pCO_2, the original experiments with fixed and dynamic ligands were repeated with Southern Hemisphere surface iron flux increased or decreased by 20× [2]. With both fixed and dynamic ligands, lowering iron input to the Southern Ocean only slightly increases the baseline atmospheric pCO_2 (<1μatm, Figs. S1a and S3a) at the control strength of Southern Ocean upwelling (20.0 Sv), since upwelling from the Deep Atlantic, via the Deep Southern Ocean, is the dominant supply of iron to the surface

of the Southern Ocean. Surface input only accounts for a small fraction of iron requirements. Alternatively, increasing iron input to the Surface Southern Ocean draws atmospheric pCO_2 down by an additional 10 and 15μatm in fixed and varying ligand models, respectively. Enhanced iron input partially relieves Southern Ocean iron limitation, increases local macronutrient uptake (Figs. S2g–i and S4g–i), and results in greater Southern Ocean export production (Figs. S1c–e and S3c–e). These anomalies are slightly compensated by a decline in production in the macronutrient-limited Surface Atlantic due to lower nitrate supply (Figs. S2a–c and S4a–c). Net ocean carbon storage is 50% greater in the dynamic ligand case since additional biological activity in the Southern and Pacific Oceans generates additional ligands (Fig. S4, right column) that can support a greater iron standing stock, leading to further export production, more complete nutrient usage, and carbon uptake from the atmosphere [4]. Other limiting factors in the Southern Ocean, such as low light levels, prevent significantly higher ligand and iron concentrations, leading to further CO_2 uptake. The sensitivity of atmospheric pCO_2 to MOC strength is similar between the control and enhanced iron input experiments (Figs. S1a and S3a). At strong rates of Southern Ocean upwelling, there is little change in relative ocean carbon outgassing with Southern Hemisphere iron deposition changes. For the fixed ligand simulations there are compensating changes in production and export between the Southern Hemisphere and Northern Hemisphere boxes: when iron is added to the Southern Ocean, relieving micronutrient limitation and consuming macronutrients and inorganic carbon, this reduces the macronutrient supply delivered to the Surface Atlantic and Pacific Oceans by the MOC, causing elevated macronutrient limitation and reduced productivity, and vice versa (Figs. S1 and S2). The surface ocean is globally macronutrient-deficient in the dynamic ligand experiments at high MOC (Fig. S4, left column). Hence, an increase in iron deposition makes little difference, although Southern Ocean production tails off somewhat due to light limitation before complete macronutrient consumption is possible, reducing the relative uptake of atmospheric CO_2 compared to the standard dust forcing. Reducing Surface Southern Ocean iron input does not change atmospheric CO_2 significantly at low MOC rates because both cases are already limited by iron: in the fixed ligand simulations, upwelling is the primary iron source with surface input again only satisfying a small proportion of iron requirements, while in the dynamic ligand simulations, there is global iron scarcity due to lack of complexing ligands (Fig. S4, middle and right columns), so reducing surface iron input makes no appreciable impact to iron limitation. There is, however, a noticeable 10 μatm increase in ocean carbon storage when iron input is increased at low rates of Southern Ocean upwelling in both fixed (enhanced CO_2 uptake, Fig. S1a) and dynamic (reduced

CO_2 outgassing, Fig. S3a) ligand models. Elevated Southern Hemisphere surface iron deposition becomes a significant iron source due to low rates of upwelling and partially relieves Southern Ocean iron limitation, which enables further macronutrient consumption (Figs. S4g and S4g) as hypothesized. Overall, the dynamic ligand feedback is the primary factor affecting atmospheric CO_2 levels, with the "iron hypothesis" [1] playing a secondary, additional role in sluggish MOC scenarios.

Depth of the Surface/Deep Atlantic interface

Paleoclimate observations and simulations provide inconclusive evidence regarding the strength and depth-extent of the AMOC during the last glacial maximum [e.g. 5–12]. The standard simulations have a fixed interface between the surface and deep Atlantic of 100 m, equivalent to the depth of the euphotic zone. This depth could also be interpreted as the depth of the AMOC maximum since the southward return flow in this model occurs in the Deep Atlantic box.

Increasing the depth of the interface to 2000 m at the control Southern Ocean upwelling strength of 20.0 Sv has only a minor effect of a 5 μatm decrease of atmospheric CO_2 with fixed ligand concentrations (Fig. S5a) and a 1 μatm increase with atmospheric CO_2 with dynamic ligand concentrations (Fig. S7a). For the fixed ligand simulations, there is an increase in the concentration of iron in the Deep Atlantic (Fig. S6e), where 51% reduced volume overcompensates the 49% decreased residence time (655 years at 20.0 Sv with the AMOC interface at 100 m to 336 years at 20.0 Sv with a deeper 2000 m interface) to enhance the accumulation of hydrothermal iron, as well as iron delivered from the Surface Atlantic in the AMOC. In contrast, Deep Atlantic macronutrient concentration declines slightly (Fig. S6d) due to fractionally lower Surface Atlantic export production (Fig. S5c). More Deep Atlantic iron is upwelled via the Southern Ocean (Fig. S6h and k), which partially alleviates iron limitation there and enables slightly enhanced biological activity (Fig. S5d), more complete macronutrient consumption (Fig. S6g), and ocean carbon uptake (Fig. S5d). Slightly reduced macronutrient export to the Surface Atlantic reduces biological activity there. With variable ligands, export production in the Surface Southern Ocean is somewhat lower, while production in the Surface Atlantic is slightly larger with a 2000 m deep Surface Atlantic box, compared to a 100 m deep Surface Atlantic box (Fig. S7c and d). Despite this, the considerably increased residence time in the upper ocean (17 years at 20.0 Sv with the AMOC interface at 100 m to 336 years at 20 Sv with a deeper 2000 m interface) causes the majority of ligands produced there to be degraded locally, which limits the accumulation of excess iron (Fig. S8b and c). The Deep Atlantic continues to accrue hydrothermal iron, but eliminating the source of excess iron delivered to the

Deep Atlantic via the AMOC results in slightly reduced iron concentrations despite marginally elevated ligand concentrations from the export production remineralization source (Fig. S8e and f). There is a slightly lower supply of iron downstream to be upwelled into the iron-limited Surface Southern Ocean (Fig. S8h and k), a lower rate of biological activity (Fig. S7d), less-complete macronutrient usage (Fig. S8g), and lower ocean carbon uptake (Fig. S7a). The slight enhancement of macronutrient delivery from the iron-limited surface boxes to the Surface Atlantic increases biological activity.

The overall sensitivity of atmospheric CO_2 across a range of Southern Ocean upwelling strengths is somewhat lower with a 2000 m deep compared to a 100 m deep Surface Atlantic (Figs. S5a and S7a). At low MOC strength, less than $\sim$20.0 Sv, changing the depth of the Atlantic Ocean interface does little to affect the change in atmospheric pCO_2 with fixed or variable ligand concentrations because the different boxes are increasingly isolated and exchange of iron and macronutrients between the three basins (particularly, from the Atlantic to the Southern and Pacific Oceans) is less important than the local sources and sinks. Alternatively, at strong MOC values greater than 25.0 Sv there is up to $\sim$20 μatm less net ocean CO_2 outgassing with fixed ligand concentrations and up to $\sim$30 μatm reduced net ocean CO_2 uptake with dynamic ligand concentrations. Both ligand scenarios see an increase in Surface Atlantic production at high MOC (Figs. S5c and S7c) due to increased residence times relative to the simulations with a shallow Surface Atlantic box (8 years at 40 Sv with the AMOC interface at 100 m to 168 years at 40 Sv with the deeper interface) and associated aeolian iron accumulation, which directly translates into enhanced nutrient and inorganic uptake (Fig. S6a and b) and atmospheric CO_2 drawdown for fixed ligand concentrations since the Surface Atlantic is iron-limited at high MOC rates. Stronger transport and upwelling of remineralized iron from the Deep Atlantic to the Southern and Pacific Oceans (Fig. S6, middle column) leads to further productivity (Fig. S5d and e), increases the completeness of nutrient usage (Fig. S6, left column), and increases ocean carbon uptake (a reduction in overall ocean outgasing). Reduced macronutrient supply from the Southern and Pacific Oceans to the Atlantic limits its further enhancement in biological activity and ocean carbon uptake. With variable ligand concentrations, longer surface Atlantic residence times again allow increased surface Atlantic production (Fig. S7c) through the accumulation of surface iron input. The extent of ligand degradation, however, increases the iron scavenging loss, reduces iron export into the Deep Atlantic and eventual upwelling into the Southern and Pacific Oceans (Fig. S8, middle column), lowers net biological carbon uptake in those iron-limited regions (Fig. S7d and e), decreases the extent of macronutrient usage (Fig. S8, left column), and results in a net reduction

in ocean carbon uptake. The opposing sign of the MOC-atmospheric pCO_2 relationship remains robust to changes in the depth of the Surface Atlantic box.

Rate of mixing between boxes

The model uses prescribed transports that are tuned to recreate the oceanic ^{14}C distribution [13], which implicitly includes the effects of both advection by the MOC and fluxes due to turbulent mixing. The latter fluxes are relatively uncertain for the modern ocean and may have been more intense during glacial times due to enhanced tidal mixing [e.g. 14]. An additional exchange of 3.0 Sv ($\sim$15% of control Southern Ocean upwelling strength) between adjacent boxes was applied. Stronger mixing increases the connection between the deep ocean and the surface and the surface Southern Ocean with the Atlantic and Pacific Oceans. For both fixed and variable ligand concentrations, atmospheric pCO_2 at the control Southern Ocean upwelling strength (20.0 Sv) is offset by $-6\,\mu$atm and $-31\,\mu$atm, respectively (Figs. S9a and S11a), as a result of globally higher biological activity (Figs. S9b–e and S11b–e). Iron in the macronutrient-limited Surface Atlantic is more depleted with increased mixing, fuelled by more macronutrient supply from the Deep Atlantic and Surface Southern Ocean compared to the standard transports alone (Figs. S10a and b, and S12a and b). Similarly, nitrate in the iron-limited Surface Southern and Pacific oceans (Figs. S10g and m, and S12g and m) is more extensively consumed due to greater upwelling and lateral iron supply. The greater nutrient consumption is enhanced with variable ligand concentrations since higher biological activity in the surface ocean generates more ligands that can support a greater iron concentration and increase production (Fig. S12, right column).

Under a weakened MOC, the mixing fluxes become an increasingly important connection between boxes. Again, enhanced net exchange of macronutrients from the Southern Ocean to the nutrient-limited Surface Atlantic Ocean and a reciprocal net flux of iron to the iron-limited Southern Ocean, as well as upwelling from the deep to the surface, enhances biological production and increases the uptake of atmospheric CO_2 compared to the standard simulations by $\sim$18 μatm in the fixed ligand experiments, while reducing the outgassing by $\sim$12 μatm in the variable ligand experiments (Figs. S9 and S11). Notably, the ligand and iron concentrations are significantly higher in the variable ligand simulations compared to the ligand- and iron-deficient standard transport simulations. At strong overturning rates, the increased connection between the deep ocean and the surface again enhances the delivery of macronutrients, micronutrients, and inorganic and increases outgassing by $<1\,\mu$atm in the fixed ligand experiments, particularly in the now iron-limited Surface Atlantic Ocean

(Fig. S10b), while net atmospheric CO_2 uptake is reduced in the variable ligand experiments due to high ligand and iron concentrations resulting in macronutrient limitation in the Surface Southern and Pacific Oceans (Fig. S12g and m) earlier than with the standard transports. Thus, the enhancement of ocean carbon drawdown peaks at 22 μatm around 35.0 Sv Southern Ocean upwelling, and then declines to $\sim$3 μatm because complete consumption of all upwelled inorganic and nutrients indicates no further atmospheric CO_2 drawdown is possible (189 μatm is close to the lower bound of atmospheric pCO_2 calculated in Ref. [15]). Again, the opposing sign of the MOC-atmospheric pCO_2 relationship remains robust to changes in the mixing rate but is more sensitive to this parameter than for surface iron input and depth of the Surface Atlantic box.

Maximum rate of biological activity (dynamic ligand model only)

The maximum rate of biological production associated with the availability of macro- and micronutrients is a crucial factor in driving the production of ligands and, therefore, the overall availability of dissolved iron. When the maximum biological production rate is perturbed by $\pm$50%, there is no real change in atmospheric CO_2 at the control strength of Southern Ocean upwelling (Fig. S11a), indicating that the control run is producing emergent biological activity rates and concentration distributions based on nutrient limitation rather than artificially limited by parameter constraints. Changes in nitrate concentrations in the macronutrient-limited Surface Atlantic and iron concentrations in the micronutrient-limited Surface Southern Ocean are negligible (Fig. S12a and h). At low MOC, the extreme global iron limitation due to low ligand abundance stifles any fundamental changes in productivity. However, approaching MOC perturbations of double the control MOC (i.e., Southern Ocean upwelling strength around 40.0 Sv), atmospheric CO_2 anomalies are slightly sensitive to a decrease in the maximum biological production rate parameter, where ligand production and iron abundance are lower, and surface nutrients are not as completely consumed. Increasing the rate by 50% produces similar atmospheric CO_2 levels as the non-perturbed simulations since surface macronutrient concentrations are already almost wholly consumed (Fig. S12, left column), and biological production is globally resource-limited.

Ligand lifetime (dynamic ligand model only)

For simulations with variable ligand concentrations, the average ligand lifetime is vital in determining changes in atmospheric pCO_2. The variable ligand ensemble was repeated with a 25% increase or decrease in degradation timescale ($1/\lambda = 283.0 \pm 71.0$ years). These values are consistent with the O(100) year residence time of ligands in deep waters of the Atlantic [upper bound, 779–1039 years, 16, 17] and partially-labile and semi-refractory

dissolved organic carbon [18]. At the control strength of Southern Ocean upwelling (20.0 Sv), changing ligand lifetime results in a 23 μatm outgassing with shorter-lived-ligands, while longer-lived ligands increase ocean carbon uptake by 24 μatm (Fig. S15a). Higher ligand lifetime increases the model parameter γ/λ from the data-optimized value of 4398.0 s to 5497.5 s, shifting the system towards the "iron-replete, macronutrient limited" regime [4], accumulating a large pool of ligands (Fig. S16, right column) due to slower rates of decay. Longer-lived ligands support a greater availability of iron (Fig. S16, middle column), resulting in greater drawdown of macronutrients (Fig. S16, left column), particularly in the iron-limited Surface Southern and Pacific Oceans. In contrast, decreasing ligand lifetime lowers the model value of γ/λ from the data-optimized value of 4398.0 s to 3298.5 s, shifting the system towards the "iron-limited, macronutrient replete" regime [4]. More rapid ligand turnover reduces ligand standing stock and lowers the overall availability of iron, resulting in more incomplete macronutrient consumption in the Surface Southern and Pacific Oceans.

At low rates of Southern Ocean upwelling, there is a minor difference in net atmospheric pCO_2 change between increased, decreased, and standard ligand lifetimes because ligand concentrations and iron levels are generally deficient, associated with reduced upwelling and limited surface ocean biological activity to generate ligands in the first place. Furthermore, higher residence times in each box allow for considerable ligand degradation, reducing export to other ocean basins. On the other hand, at high rates of Southern Ocean upwelling, the differences in atmospheric pCO_2 for increased and decreased ligand lifetimes are more significant, up to 33 μatm more uptake and up to 48 μatm more outgassing, respectively, compared to the standard case. Shorter residence times due to rapid circulation enable longer-lived ligands to be widely redistributed and to support a greater pool of dissolved iron, which allows more complete consumption of macronutrients in the Surface Southern and Pacific Oceans. Reduced macronutrient supply from the Southern and Pacific Oceans to the Surface Atlantic decreases biological activity there. Increased production generates a more significant concentration of ligands, reinforcing iron availability and macronutrient and inorganic drawdown. Indeed, like the increased mixing simulations, there are enough ligands and iron at the Southern Ocean upwelling strengths between 30.0–40.0 Sv to almost wholly deplete macronutrient concentrations in the Surface Southern and Pacific Oceans, leading to no further atmospheric CO_2 drawdown. In contrast, shorter-lived ligands support a smaller iron pool, which reduces the extent of macronutrient consumption in the Surface Southern and Pacific Oceans, resulting in an increase in ocean carbon outgassing compared to the standard ligand lifetime. Greater supply of nitrate to the Surface Atlantic increases biological activity there. Net decline in global ligand

stocks, reduced iron availability, and lower total export production generate fewer ligands, which leads to further enhanced ocean carbon outgassing. Overall, the opposite direction of the MOC-atmospheric pCO_2 relationship with dynamic ligand concentration remains robust but is more sensitive (i.e., larger gradient) with higher ligand lifetime and less sensitive (i.e., smaller gradient) with lower ligand lifetime.

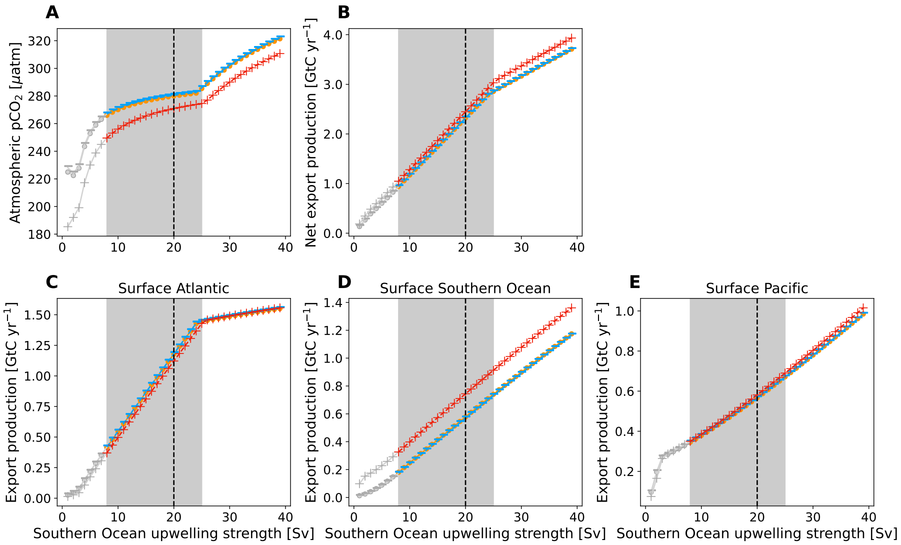

Fig. S1 Model sensitivity to Southern Hemisphere surface iron input with fixed, uniform ligand concentrations. Steady-state results of Meridional Overturning Circulation strength experiments for (a) atmospheric pCO$_2$ (μatm), (b) globally-integrated export production (GtC yr^{-1}), and (c–e) local export production (GtC yr^{-1}) in the Surface Atlantic, Surface Southern Ocean, and Surface Pacific boxes, respectively. The standard simulation (circle symbol, orange) is perturbed with a 20× increase (plus symbols, red) or decrease (dash symbol, blue) in Southern Hemisphere surface iron input. The vertical dashed line indicates the control Southern Ocean upwelling (20.0 Sv). The grey box highlights the realistic range of AMOC transport for the last glacial maximum [8.0–25.0 Sv, e.g. 9, 10, 12, 19, 20].

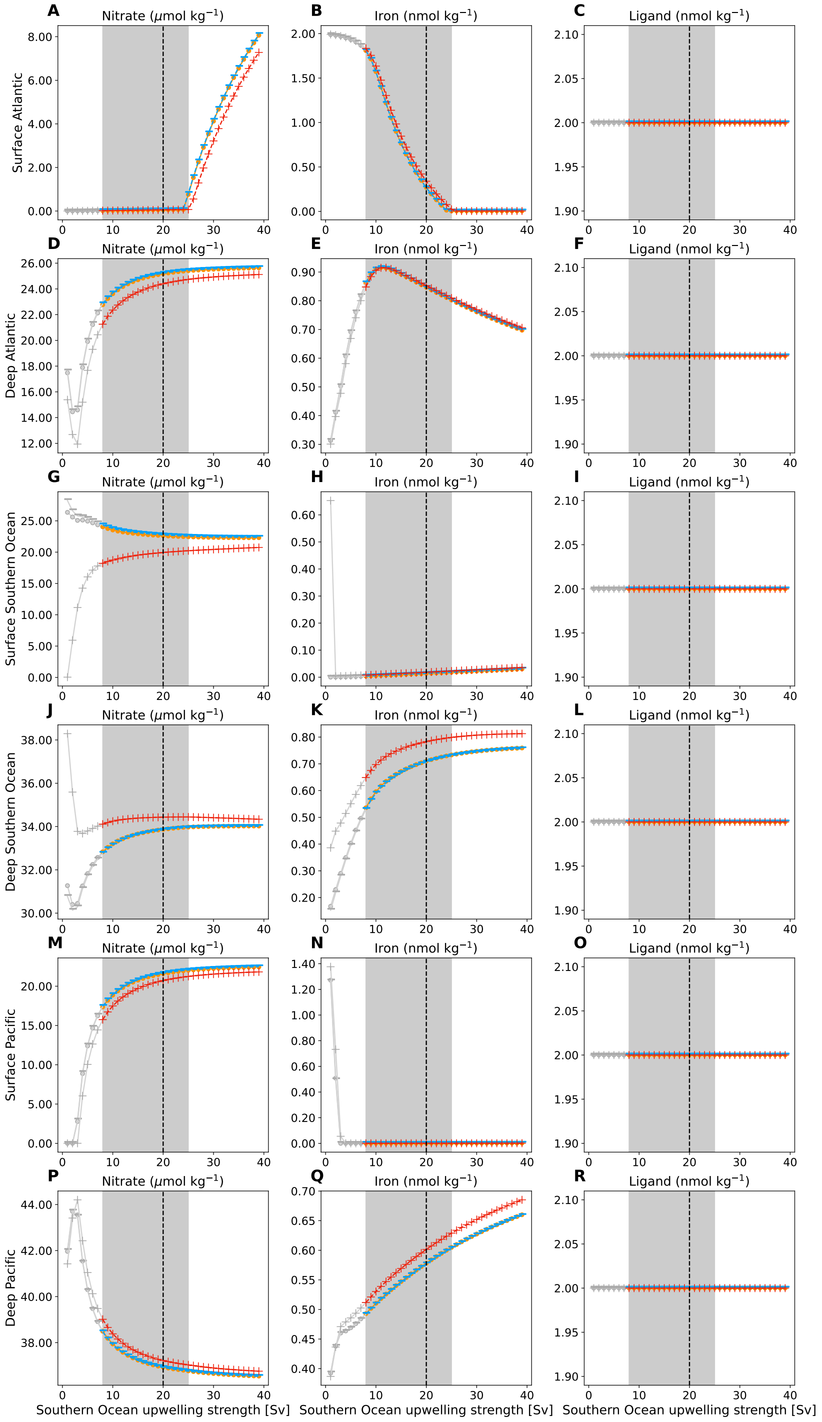

Fig. S2 Model sensitivity to Southern Hemisphere surface iron input with fixed, uniform ligand concentrations. Steady-state results of Meridional Overturning Circulation strength experiments for nitrate (μmol N kg^{-1}, left column), iron (nmol Fe kg^{-1}, middle column), and ligands (nmol L kg^{-1}, right column) in (a–c) the Surface Atlantic, (d–f) the Deep Atlantic, (g–i) the Surface Southern Ocean, (j–l) the Deep Southern Ocean, (m–o) the Surface Pacific, and (p–r) the Deep Pacific Ocean. The standard simulation (circle symbol, orange) is perturbed with a 20× increase (plus symbols, red) or decrease (dash symbol, blue) in Southern Hemisphere surface iron input. The vertical dashed line indicates the control Southern Ocean upwelling (20.0 Sv). The grey box highlights the realistic range of AMOC transport for the last glacial maximum [8.0–25.0 Sv, e.g. 9, 10, 12, 19, 20].

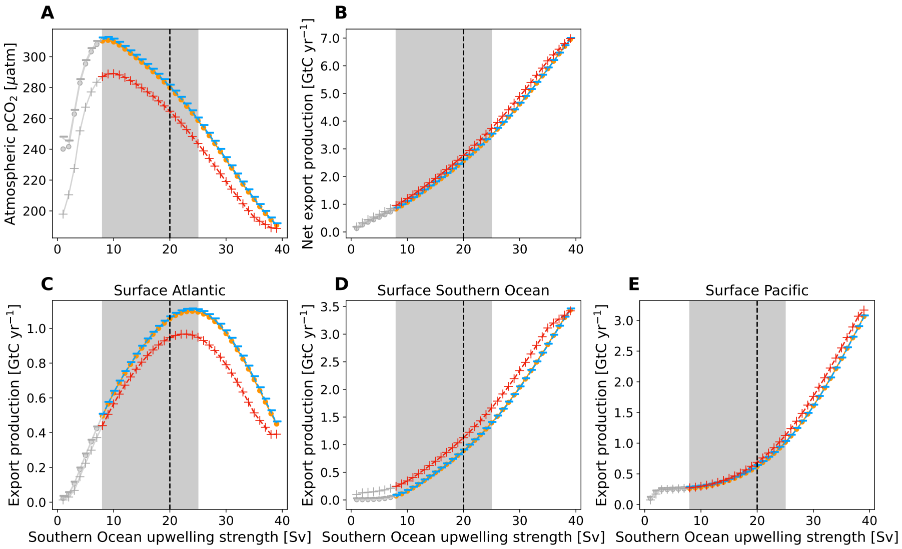

Fig. S3 Model sensitivity to Southern Hemisphere surface iron input with dynamic ligand concentrations. Steady-state results of Meridional Overturning Circulation strength experiments for (a) atmospheric pCO_2 (μatm), (b) globally-integrated export production (GtC yr^{-1}), and (c–e) local export production (GtC yr^{-1}) in the Surface Atlantic, Surface Southern Ocean, and Surface Pacific boxes, respectively. The standard simulation (circle symbol, orange) is perturbed with a 20× increase (plus symbols, red) or decrease (dash symbol, blue) in Southern Hemisphere surface iron input. The vertical dashed line indicates the control Southern Ocean upwelling (20.0 Sv). The grey box highlights the realistic range of AMOC transport for the last glacial maximum [8.0–25.0 Sv, e.g. 9, 10, 12, 19, 20].

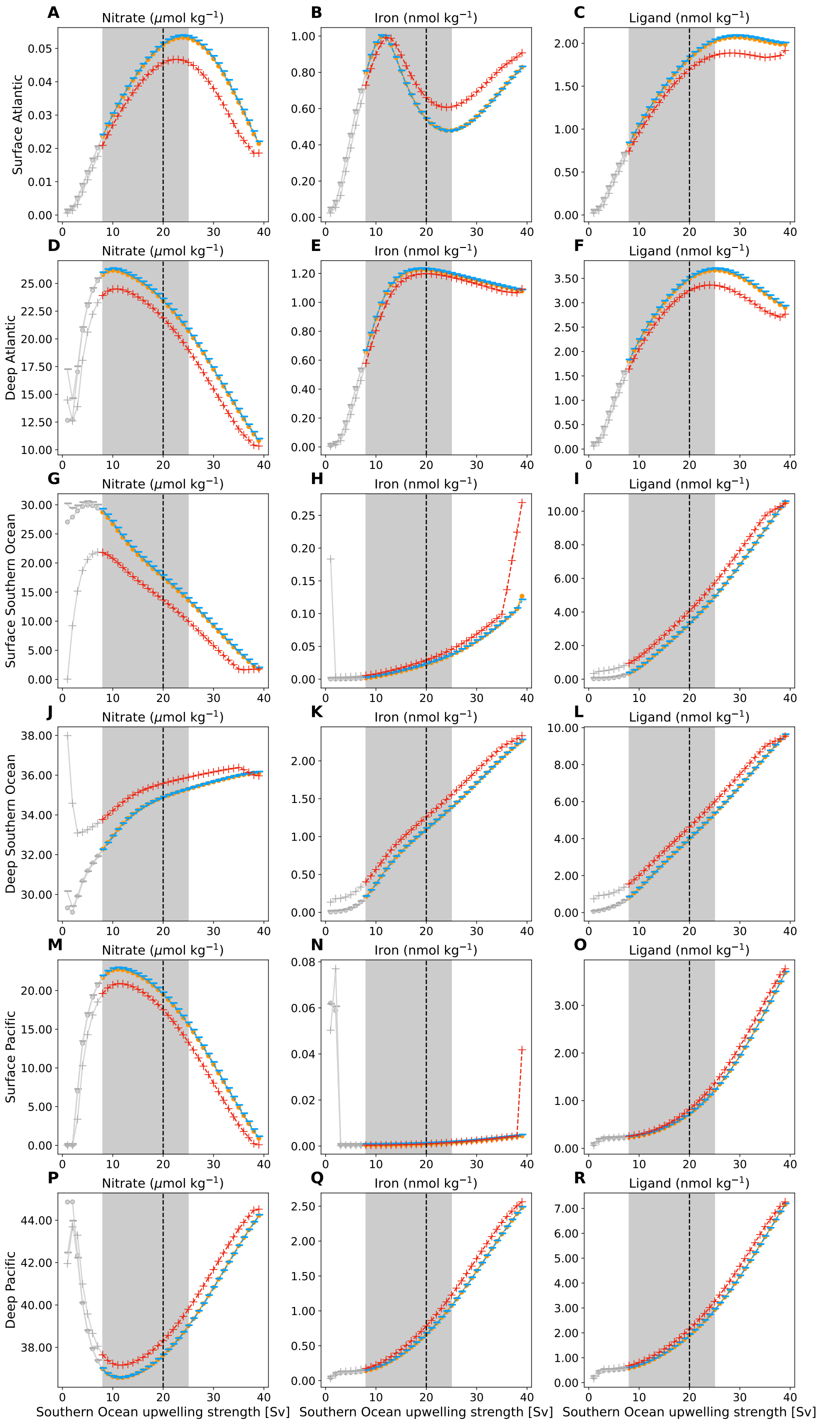

Fig. S4 Model sensitivity to Southern Hemisphere surface iron input with dynamic ligand concentrations. Steady-state results of Meridional Overturning Circulation strength experiments for nitrate (μmol N kg^{-1}, left column), iron (nmol Fe kg^{-1}, middle column), and ligands (nmol L kg^{-1}, right column) in (a–c) the Surface Atlantic, (d–f) the Deep Atlantic, (g–i) the Surface Southern Ocean, (j–l) the Deep Southern Ocean, (m–o) the Surface Pacific, and (p–r) the Deep Pacific Ocean. The standard simulation (circle symbol, orange) is perturbed with a 20$\times$ increase (plus symbols, red) or decrease (dash symbol, blue) in Southern Hemisphere surface iron input. The vertical dashed line indicates the control Southern Ocean upwelling (20.0 Sv). The grey box highlights the realistic range of AMOC transport for the last glacial maximum [8.0–25.0 Sv, e.g. 9, 10, 12, 19, 20].

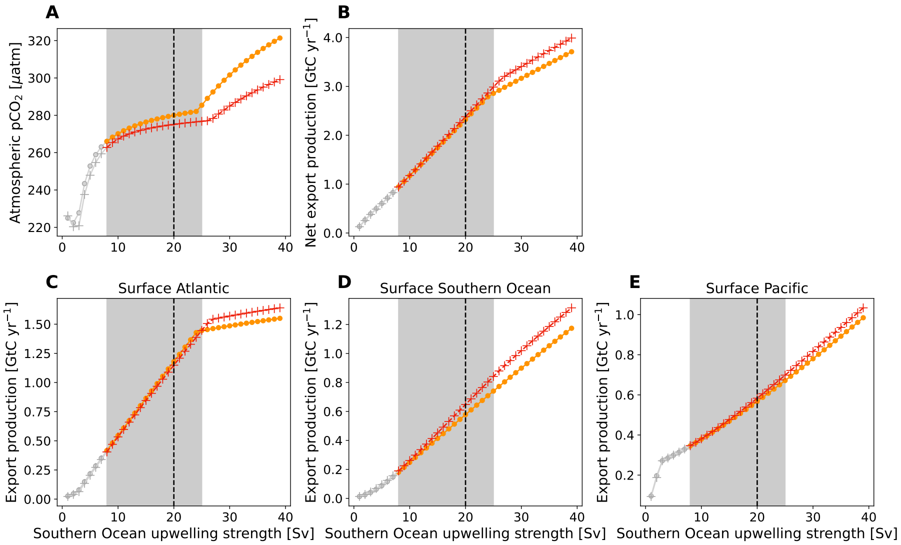

Fig. S5 Model sensitivity to surface/deep Atlantic interface depth with fixed, uniform ligand concentrations. Steady-state results of Meridional Overturning Circulation strength experiments for (a) atmospheric pCO_2 (μatm), (b) globally-integrated export production (GtC yr^{-1}), and (c–e) local export production (GtC yr^{-1}) in the Surface Atlantic, Surface Southern Ocean, and Surface Pacific boxes, respectively. The standard simulation (circle symbol, orange) is perturbed with an increase in the depth of the interface between surface and deep Atlantic boxes (from 100 to 2000 m, plus symbols, red). The vertical dashed line indicates the control Southern Ocean upwelling (20.0 Sv). The grey box highlights the realistic range of AMOC transport for the last glacial maximum [8.0–25.0 Sv, e.g. 9, 10, 12, 19, 20].

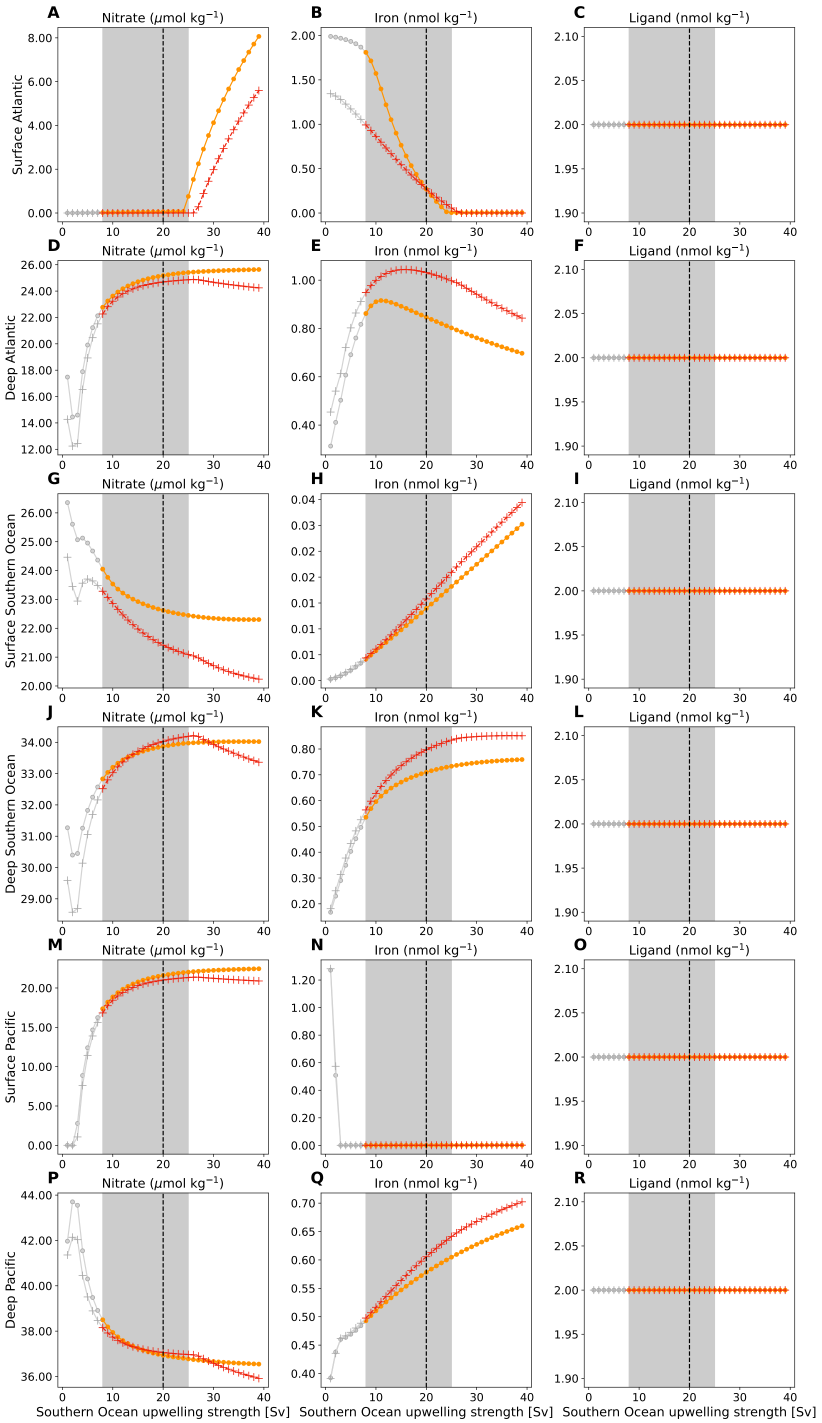

Fig. S6 Model sensitivity to surface/deep Atlantic interface depth with fixed, uniform ligand concentrations. Steady-state results of Meridional Overturning Circulation strength experiments for nitrate (μmol N kg^{-1}, left column), iron (nmol Fe kg^{-1}, middle column), and ligands (nmol L kg^{-1}, right column) in (a–c) the Surface Atlantic, (d–f) the Deep Atlantic, (g–i) the Surface Southern Ocean, (j–l) the Deep Southern Ocean, (m–o) the Surface Pacific, and (p–r) the Deep Pacific Ocean. The standard simulation (circle symbol, orange) is perturbed with an increase in the depth of the interface between surface and deep Atlantic boxes (from 100 to 2000 m, plus symbols, red). The vertical dashed line indicates the control Southern Ocean upwelling (20.0 Sv). The grey box highlights the realistic range of AMOC transport for the last glacial maximum [8.0–25.0 Sv, e.g. 9, 10, 12, 19, 20].

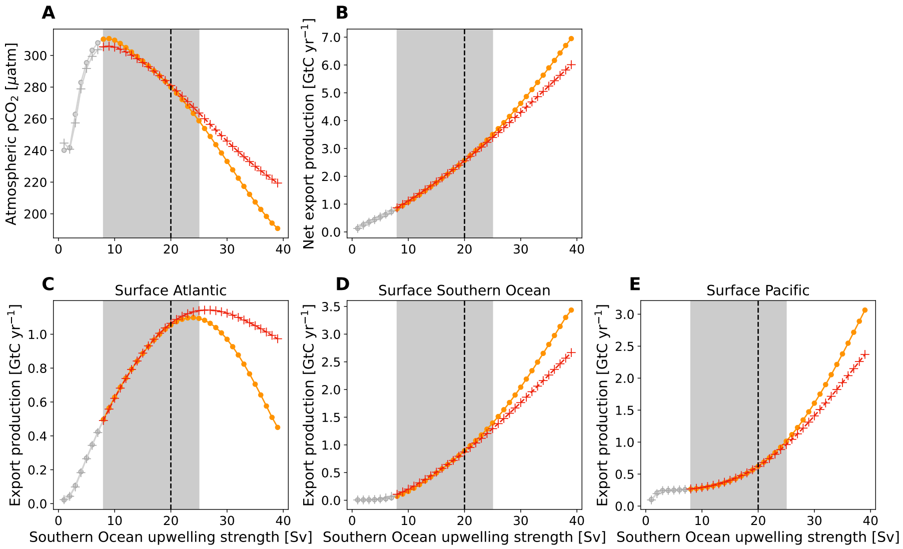

Fig. S7 Model sensitivity to surface/deep Atlantic interface depth with dynamic ligand concentrations. Steady-state results of Meridional Overturning Circulation strength experiments for (a) atmospheric pCO_2 (μatm), (b) globally-integrated export production (GtC yr^{-1}), and (c–e) local export production (GtC yr^{-1}) in the Surface Atlantic, Surface Southern Ocean, and Surface Pacific boxes, respectively. The standard simulation (circle symbol, orange) is perturbed with an increase in the depth of the interface between surface and deep Atlantic boxes (from 100 to 2000 m, plus symbols, red). The vertical dashed line indicates the control Southern Ocean upwelling (20.0 Sv). The grey box highlights the realistic range of AMOC transport for the last glacial maximum [8.0–25.0 Sv, e.g. 9, 10, 12, 19, 20].

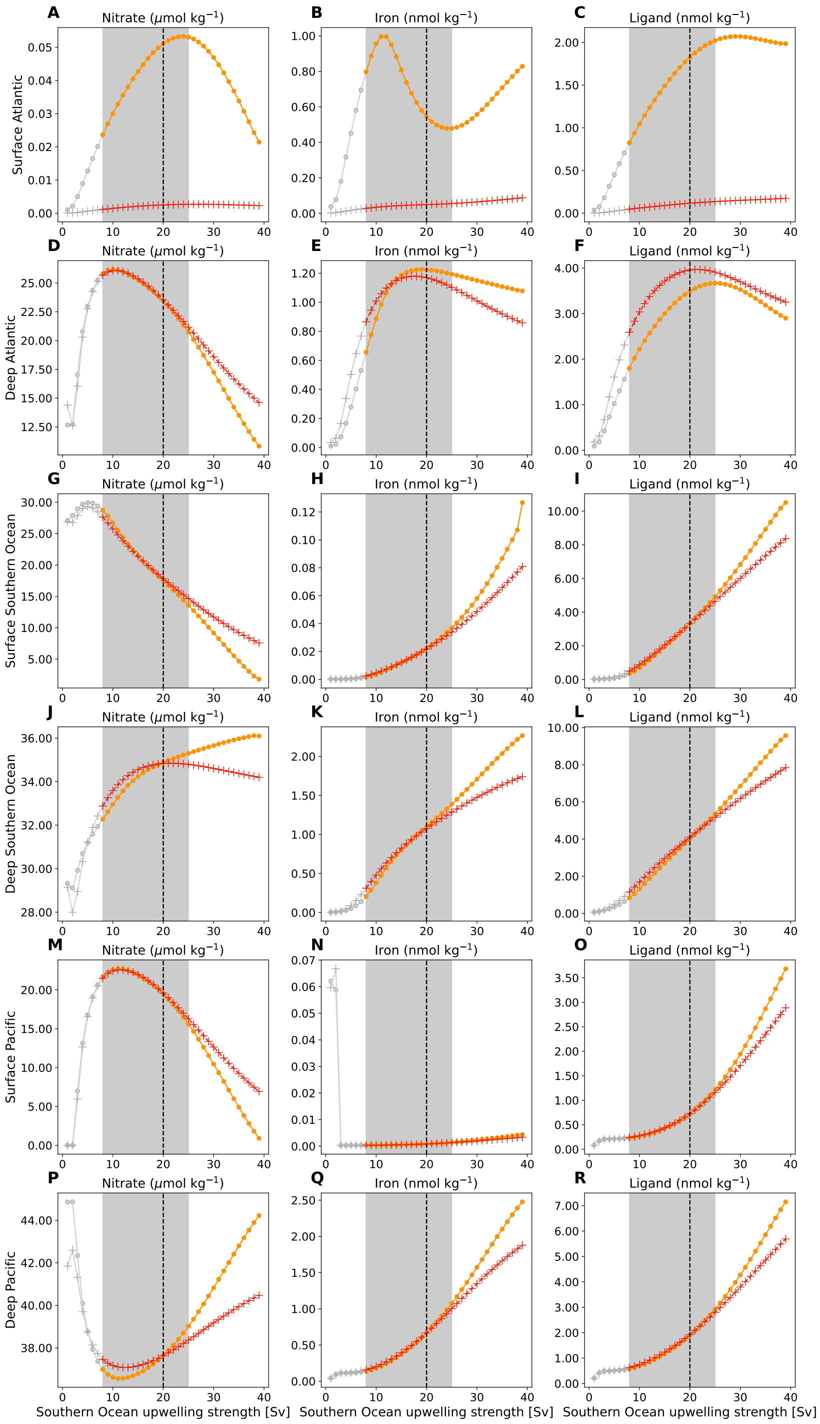

Fig. S8 Model sensitivity to surface/deep Atlantic interface depth with dynamic ligand concentrations. Steady-state results of Meridional Overturning Circulation strength experiments for nitrate (μmol N kg^{-1}, left column), iron (nmol Fe kg^{-1}, middle column), and ligands (nmol L kg^{-1}, right column) in (a–c) the Surface Atlantic, (d–f) the Deep Atlantic, (g–i) the Surface Southern Ocean, (j–l) the Deep Southern Ocean, (m–o) the Surface Pacific, and (p–r) the Deep Pacific Ocean. The standard simulation (circle symbol, orange) is perturbed with an increase in the depth of the interface between surface and deep Atlantic boxes (from 100 to 2000 m, plus symbols, red). The vertical dashed line indicates the control Southern Ocean upwelling (20.0 Sv). The grey box highlights the realistic range of AMOC transport for the last glacial maximum [8.0–25.0 Sv, e.g. 9, 10, 12, 19, 20].

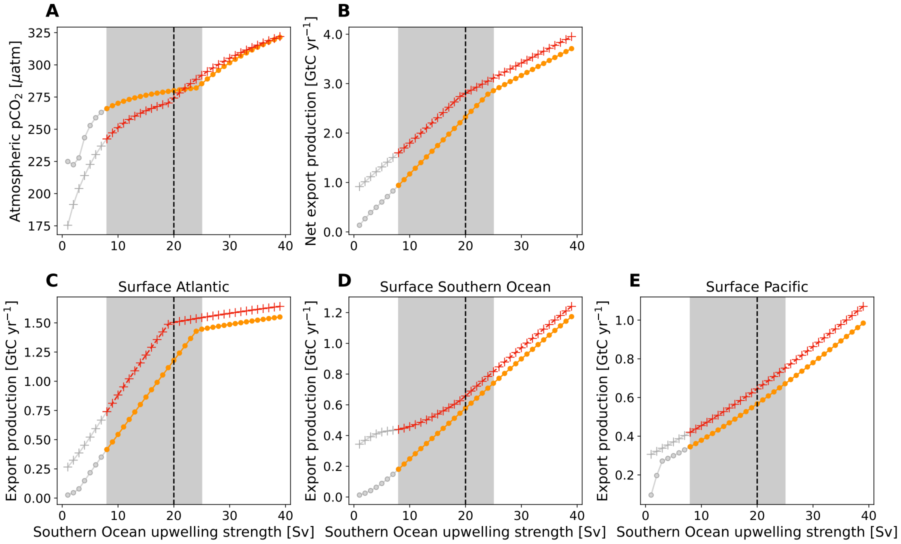

Fig. S9 Model sensitivity to diffusive mixing rates with fixed, uniform ligand concentrations. Steady-state results of Meridional Overturning Circulation strength experiments for (a) atmospheric pCO_2 (μatm), (b) globally-integrated export production (GtC yr^{-1}), and (c–e) local export production (GtC yr^{-1}) in the Surface Atlantic, Surface Southern Ocean, and Surface Pacific boxes, respectively. The standard simulation (circle symbol, orange) is perturbed with an increase in mixing fluxes between adjacent boxes (from 0.0 to 3.0 Sv, plus symbols, red). The vertical dashed line indicates the control Southern Ocean upwelling (20.0 Sv). The grey box highlights the realistic range of AMOC transport for the last glacial maximum [8.0–25.0 Sv, e.g. 9, 10, 12, 19, 20].

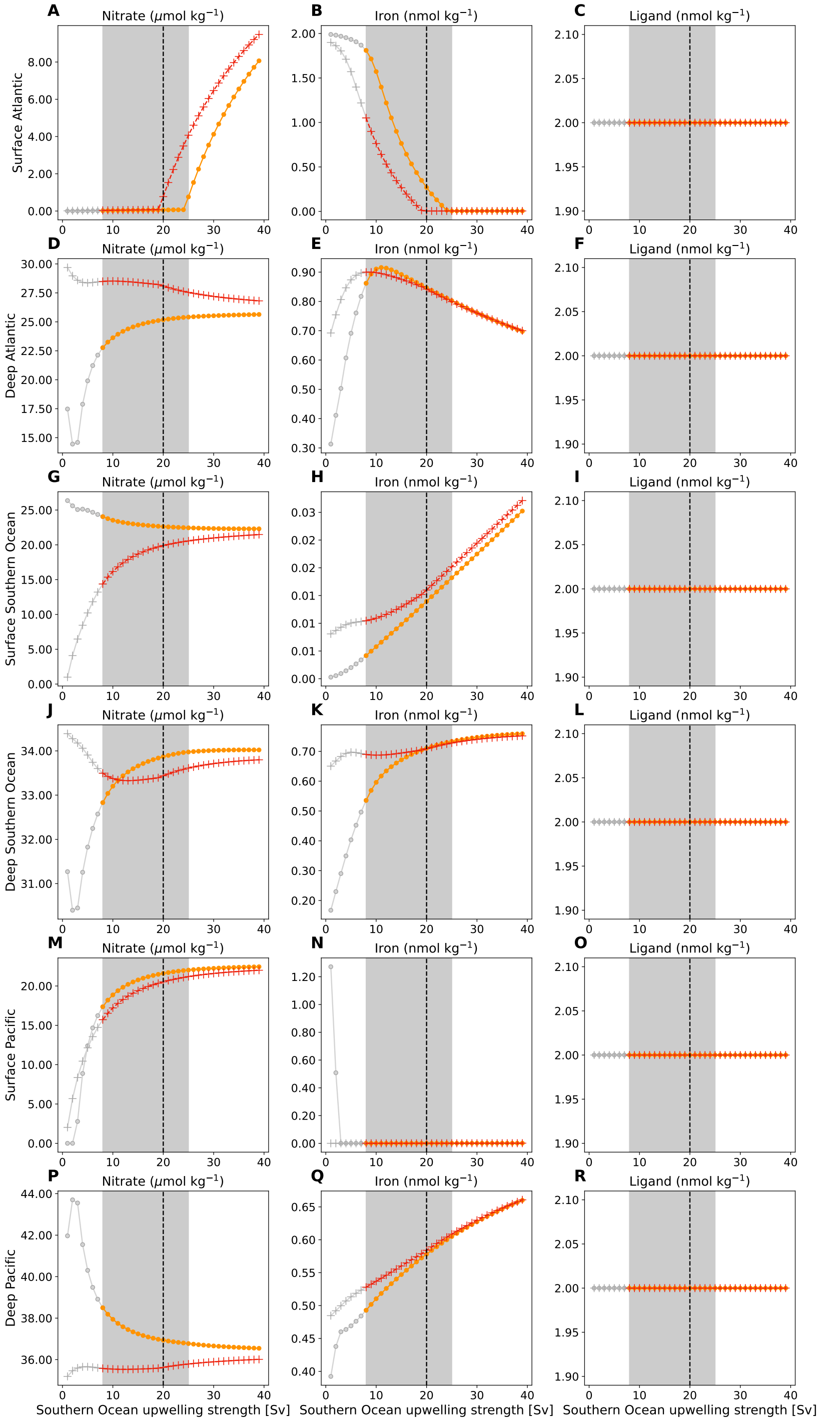

Fig. S10 Model sensitivity to diffusive mixing rates with fixed, uniform ligand concentrations. Steady-state results of Meridional Overturning Circulation strength experiments for nitrate (μmol N kg^{-1}, left column), iron (nmol Fe kg^{-1}, middle column), and ligands (nmol L kg^{-1}, right column) in (a–c) the Surface Atlantic, (d–f) the Deep Atlantic, (g–i) the Surface Southern Ocean, (j–l) the Deep Southern Ocean, (m–o) the Surface Pacific, and (p–r) the Deep Pacific Ocean. The standard simulation (circle symbol, orange) is perturbed with an increase in mixing fluxes between adjacent boxes (from 0.0 to 3.0 Sv, plus symbols, red). The vertical dashed line indicates the control Southern Ocean upwelling (20.0 Sv). The grey box highlights the realistic range of AMOC transport for the last glacial maximum [8.0–25.0 Sv, e.g. 9, 10, 12, 19, 20].

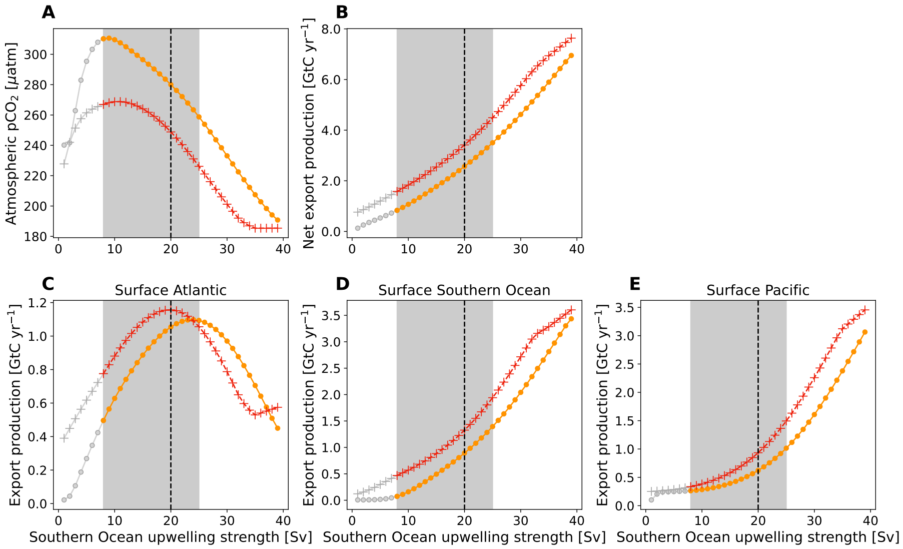

Fig. S11 Model sensitivity to diffusive mixing rates with dynamic ligand concentrations. Steady-state results of Meridional Overturning Circulation strength experiments for (a) atmospheric pCO_2 (μatm), (b) globally-integrated export production (GtC yr^{-1}), and (c–e) local export production (GtC yr^{-1}) in the Surface Atlantic, Surface Southern Ocean, and Surface Pacific boxes, respectively. The standard simulation (circle symbol, orange) is perturbed with an increase in mixing fluxes between adjacent boxes (from 0.0 to 3.0 Sv, plus symbols, red). The vertical dashed line indicates the control Southern Ocean upwelling (20.0 Sv). The grey box highlights the realistic range of AMOC transport for the last glacial maximum [8.0–25.0 Sv, e.g. 9, 10, 12, 19, 20].

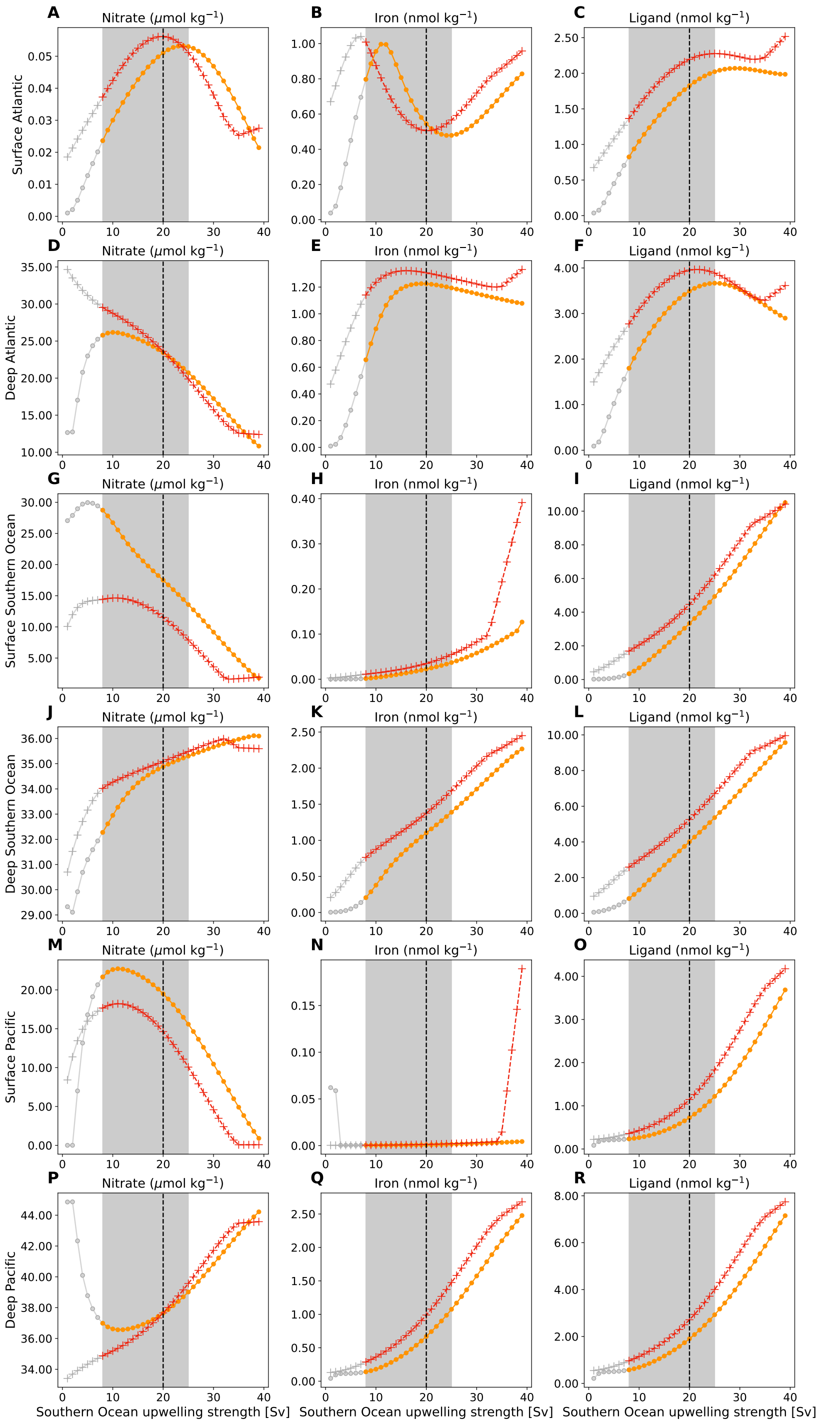

Fig. S12 Model sensitivity to diffusive mixing rates with dynamic ligand concentrations. Steady-state results of Meridional Overturning Circulation strength experiments for nitrate (μmol N kg^{-1}, left column), iron (nmol Fe kg^{-1}, middle column), and ligands (nmol L kg^{-1}, right column) in (a–c) the Surface Atlantic, (d–f) the Deep Atlantic, (g–i) the Surface Southern Ocean, (j–l) the Deep Southern Ocean, (m–o) the Surface Pacific, and (p–r) the Deep Pacific Ocean. The standard simulation (circle symbol, orange) is perturbed with an increase in mixing fluxes between adjacent boxes (from 0.0 to 3.0 Sv, plus symbols, red). The vertical dashed line indicates the control Southern Ocean upwelling (20.0 Sv). The grey box highlights the realistic range of AMOC transport for the last glacial maximum [8.0–25.0 Sv, e.g. 9, 10, 12, 19, 20].

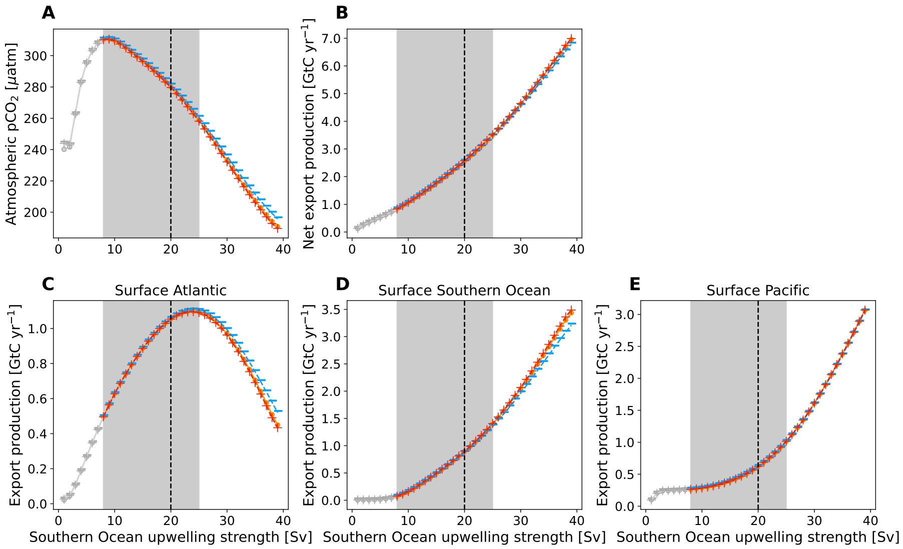

Fig. S13 Model sensitivity to biological productivity rate with dynamic ligand concentrations. Steady-state results of Meridional Overturning Circulation strength experiments for (a) atmospheric pCO_2 (μatm), (b) globally-integrated export production (GtC yr^{-1}), and (c–e) local export production (GtC yr^{-1}) in the Surface Atlantic, Surface Southern Ocean, and Surface Pacific boxes, respectively. The standard simulation (circle symbol, orange) is perturbed with a 50% increase (plus symbols, red) or decrease (dash symbols, blue) in the maximum rate of biological productivity ($6\pm3\,\mu$mol P $m^{-3}\,s^{-1}$). The vertical dashed line indicates the control Southern Ocean upwelling (20.0 Sv). The grey box highlights the realistic range of AMOC transport for the last glacial maximum [8.0–25.0 Sv, e.g. 9, 10, 12, 19, 20].

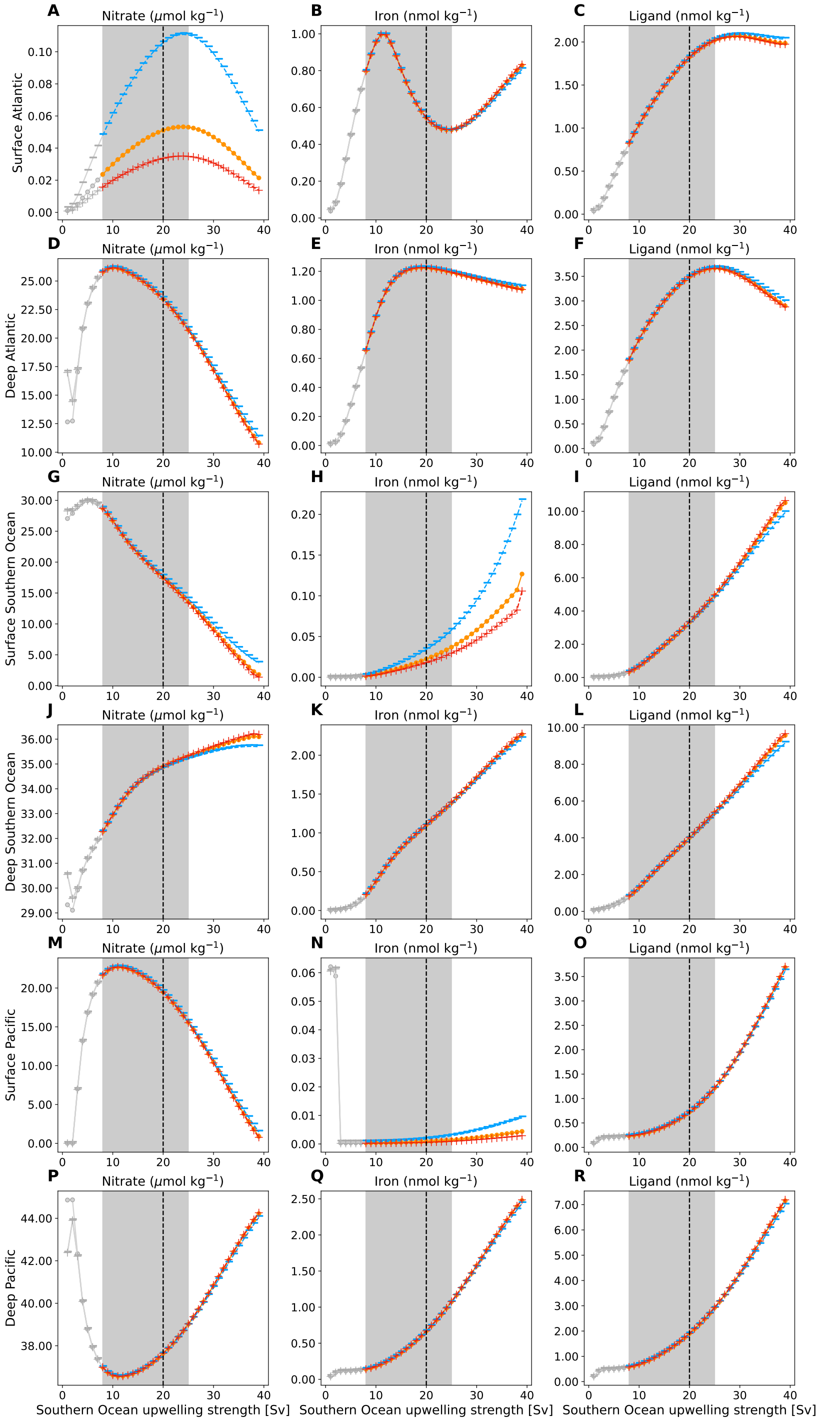

Fig. S14 Model sensitivity to biological productivity rate with dynamic ligand concentrations. Steady-state results of Meridional Overturning Circulation strength experiments for nitrate (μmol N kg^{-1}, left column), iron (nmol Fe kg^{-1}, middle column), and ligands (nmol L kg^{-1}, right column) in (a–c) the Surface Atlantic, (d–f) the Deep Atlantic, (g–i) the Surface Southern Ocean, (j–l) the Deep Southern Ocean, (m–o) the Surface Pacific, and (p–r) the Deep Pacific Ocean. The standard simulation (circle symbol, orange) is perturbed with a 50% increase (plus symbols, red) or decrease (dash symbols, blue) in the maximum rate of biological productivity ($6 \pm 3\,\mu$mol P m^{-3} s^{-1}). The vertical dashed line indicates the control Southern Ocean upwelling (20.0 Sv). The grey box highlights the realistic range of AMOC transport for the last glacial maximum [8.0–25.0 Sv, e.g. 9, 10, 12, 19, 20].

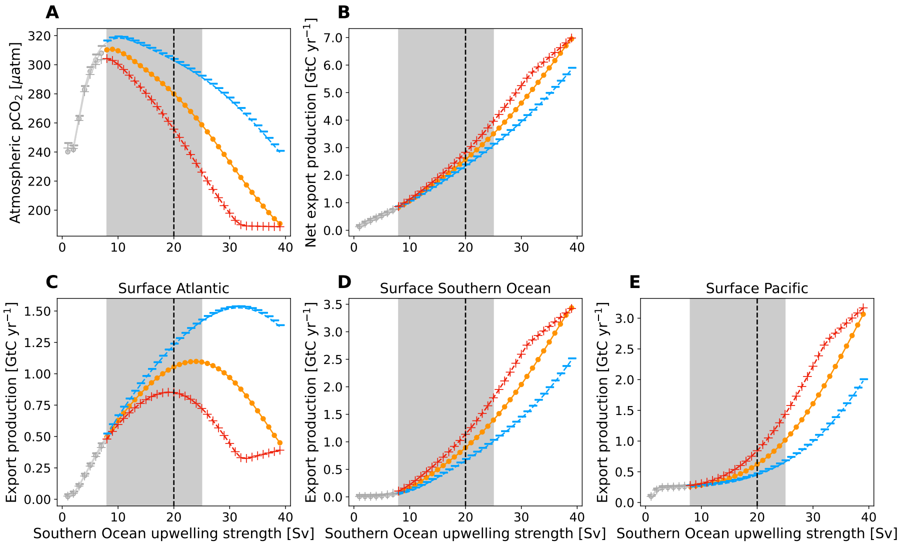

Fig. S15 Model sensitivity to ligand lifetime. Steady-state results of Meridional Overturning Circulation strength experiments with dynamic ligand concentrations for (a) atmospheric pCO_2 (μatm), (b) globally-integrated export production (GtC yr^{-1}), and (c–e) local export production (GtC yr^{-1}) in the Surface Atlantic, Surface Southern Ocean, and Surface Pacific boxes, respectively. The standard simulation (circle symbol, orange) is perturbed with a 25% increase (plus symbols, red) or decrease (dash symbols, blue) in ligand lifetime timescale (282±70 years in the deep ocean). The vertical dashed line indicates the control Southern Ocean upwelling (20.0 Sv). The grey box highlights the realistic range of AMOC transport for the last glacial maximum [8.0–25.0 Sv, e.g. 9, 10, 12, 19, 20].

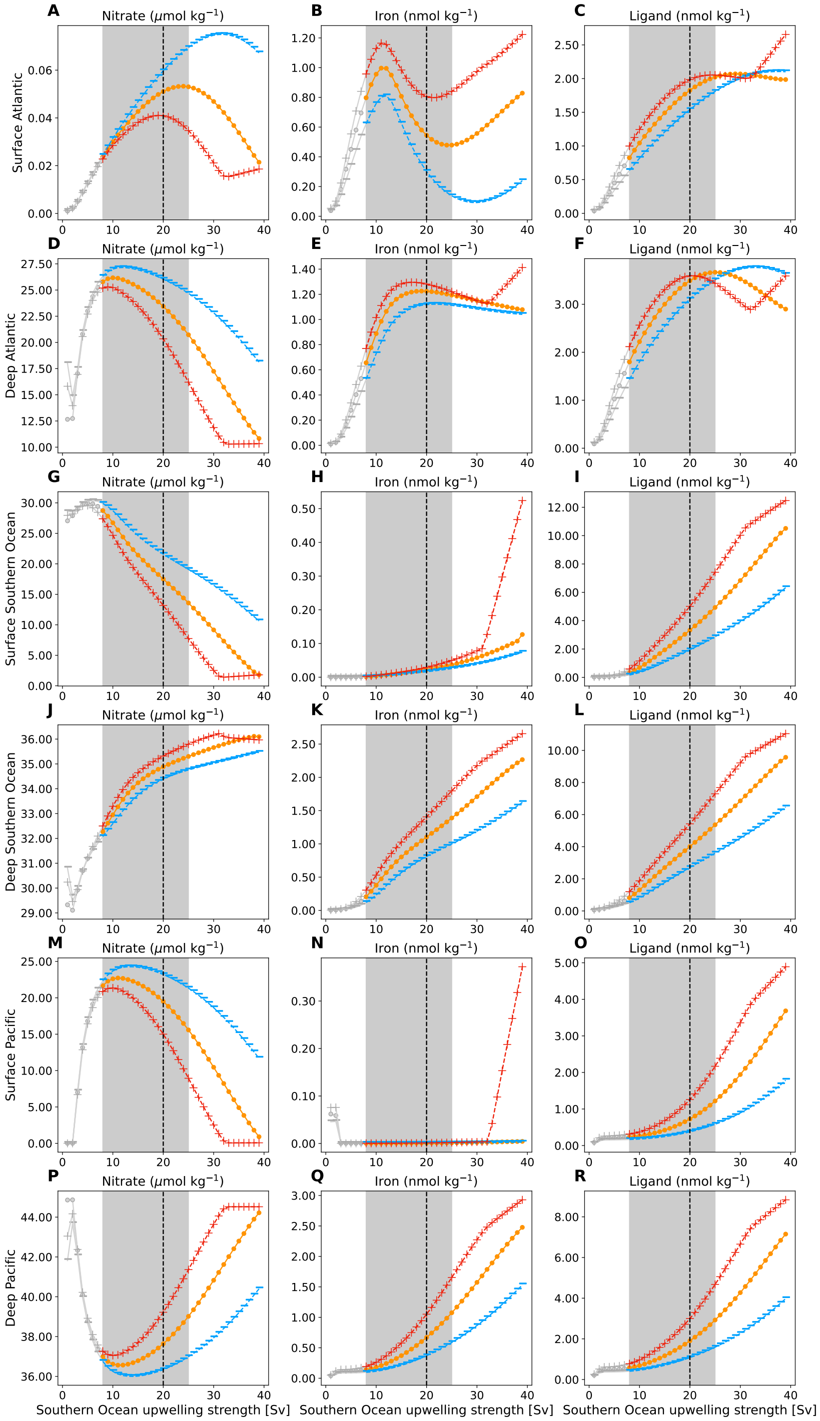

Fig. S16 Model sensitivity to ligand lifetime. Steady-state results of Meridional Overturning Circulation strength experiments with dynamic ligand concentrations for nitrate (μmol N kg^{-1}, left column), iron (nmol Fe kg^{-1}, middle column), and ligands (nmol L kg^{-1}, right column) in (a–c) the Surface Atlantic, (d–f) the Deep Atlantic, (g–i) the Surface Southern Ocean, (j–l) the Deep Southern Ocean, (m–o) the Surface Pacific, and (p–r) the Deep Pacific Ocean. The standard simulation (circle symbol, orange) is perturbed with a 25% increase (plus symbols, red) or decrease (dash symbols, blue) in ligand lifetime timescale (282$\pm$70 years in the deep ocean). The vertical dashed line indicates the control Southern Ocean upwelling (20.0 Sv). The grey box highlights the realistic range of AMOC transport for the last glacial maximum [8.0–25.0 Sv, e.g. 9, 10, 12, 19, 20].

Supplementary References

[1] Martin, J. H. Glacial–interglacial CO_2 change: the iron hypothesis. *Paleoceanography* **5**, 1–13 (1990). URL https://doi.org/10.1029/PA005i001p00001.

[2] Mahowald, N. M. *et al.* Change in atmospheric mineral aerosols in response to climate: Last glacial period, preindustrial, modern and doubled carbon dioxide climates. *J. Geophys. Res.* **111** (2006). URL https://doi.org/10.1029/2005JD006653.

[3] Parekh, P., Dutkiewicz, S., Follows, M. J. & Ito, T. Atmospheric carbon dioxide in a less dusty world. *Geophys. Res. Lett.* **33** (2006). URL https://doi.org/10.1029/2005GL025098.

[4] Lauderdale, J. M., Braakman, R., Forget, G., Dutkiewicz, S. & Follows, M. J. Microbial feedbacks optimize ocean iron availability. *Proc. Nat. Acad. Sci.* **117**, 4842 (2020). URL https://doi.org/10.1073/pnas.1917277117.

[5] Oppo, D. W. *et al.* Data Constraints on Glacial Atlantic Water Mass Geometry and Properties. *Paleoceanogr. Paleoclimatology* **33**, 1013–1034 (2018). URL https://doi.org/10.1029/2018PA003408.

[6] Menviel, L. C. *et al.* Enhanced Mid-depth Southward Transport in the Northeast Atlantic at the Last Glacial Maximum Despite a Weaker AMOC. *Paleoceanogr. Paleoclimatology* **35**, e2019PA003793 (2020). URL https://doi.org/10.1029/2019PA003793.

[7] Gu, S. *et al.* Assessing the potential capability of reconstructing glacial Atlantic water masses and AMOC using multiple proxies in CESM. *Earth and Planetary Science Letters* **541**, 116294 (2020). URL https://doi.org/10.1016/j.epsl.2020.116294.

[8] Pavia, F. J., Jones, C. S. & Hines, S. K. Geometry of the Meridional Overturning Circulation at the Last Glacial Maximum. *Journal of Climate* **35**, 5465–5482 (2022). URL https://doi.org/10.1175/JCLI-D-21-0671.1.

[9] Muglia, J. & Schmittner, A. Carbon isotope constraints on glacial Atlantic meridional overturning: Strength vs depth. *Quaternary Science Reviews* **257**, 106844 (2021). URL https://doi.org/10.1016/j.quascirev.2021.106844.

[10] Pöppelmeier, F., Jeltsch-Thömmes, A., Lippold, J., Joos, F. & Stocker, T. F. Multi-proxy constraints on Atlantic circulation dynamics since the last ice age. *Nat. Geosci.* **16**, 349–356 (2023). URL https://doi.org/10.1038/s41561-023-01140-3.

[11] Liu, Z. Evolution of Atlantic Meridional Overturning Circulation since the last glaciation: Model simulations and relevance to present and future. *Philos. Trans. R. Soc. Math. Phys. Eng. Sci.* **381**, 20220190 (2023). URL https://doi.org/10.1098/rsta.2022.0190.

[12] Kageyama, M. *et al.* The PMIP4 Last Glacial Maximum experiments: Preliminary results and comparison with the PMIP3 simulations. *Clim. Past* **17**, 1065–1089 (2021). URL https://doi.org/10.5194/cp-17-1065-2021.

[13] Broecker, W. S. & Peng, T.-H. Carbon cycle: 1985 glacial to interglacial changes in the operation of the global carbon cycle. *Radiocarbon* **28**, 309–327 (1986). URL https://doi.org/10.1017/S0033822200007414.

[14] Green, J. A. M. *et al.* Tidal mixing and the meridional overturning circulation from the last glacial maximum. *Geophys. Res. Lett.* **36** (2009). URL https://doi.org/10.1029/2009GL039309.

[15] Sarmiento, J. L. & Toggweiler, J. R. A new model for the role of the oceans in determining atmospheric pCO_2. *Nature* **308**, 621–624 (1984). URL https://doi.org/10.1038/308621a0.

[16] Gerringa, L. J. A., Rijkenberg, M. J. A., Schoemann, V., Laan, P. & de Baar, H. J. W. Organic complexation of iron in the West Atlantic Ocean. *Mar. Chem.* **177**, 434–446 (2015). URL https://doi.org/10.1016/j.marchem.2015.04.007.

[17] Buck, K. N., Sedwick, P. N., Sohst, B. & Carlson, C. A. Organic complexation of iron in the eastern tropical South Pacific: Results from US GEOTRACES Eastern Pacific Zonal Transect (GEOTRACES cruise GP16). *Mar. Chem.* **201**, 229–241 (2018). URL https://doi.org/10.1016/j.marchem.2017.11.007.

[18] Völker, C. & Tagliabue, A. Modeling organic iron-binding ligands in a three-dimensional biogeochemical ocean model. *Mar. Chem.* **173**, 67–77 (2015). URL https://doi.org/10.1016/j.marchem.2014.11.008.

[19] Muglia, J., Skinner, L. C. & Schmittner, A. Weak overturning circulation and high Southern Ocean nutrient utilization maximized glacial ocean carbon. *Earth and Planetary Science Letters* **496**, 47–56 (2018). URL https://doi.org/10.1016/j.epsl.2018.05.038.

[20] Oka, A. *et al.* Glacial mode shift of the Atlantic meridional overturning circulation by warming over the Southern Ocean. *Commun. Earth Environ.* **2**, 1–8 (2021). URL https://doi.org/10.1038/s43247-021-00226-3.
